# Targeting metabolic disturbance in the diabetic heart

**DOI:** 10.1186/s12933-015-0173-8

**Published:** 2015-02-07

**Authors:** Jesús Fuentes-Antrás, Belén Picatoste, Elisa Ramírez, Jesús Egido, José Tuñón, Óscar Lorenzo

**Affiliations:** Vascular, Renal and Diabetes Laboratory, IIS-Fundación Jiménez Díaz, Autónoma University, Av. Reyes Católicos 2, Madrid, 28040 Spain; Spanish Biomedical Research Centre in Diabetes and Associated Metabolic Disorders (CIBERDEM) network, Madrid, Spain

**Keywords:** Diabetic cardiomyopathy, Metformin, Dipeptidyl peptidase-4, Glucagon-like protein-1, Advanced glycation end-products, Statins, Peroxisome proliferator activated receptor agonists, Fatty acid translocase/cluster of differentiation-36, Toll-like receptor-4, Nod-like receptor-3

## Abstract

Diabetic cardiomyopathy is defined as ventricular dysfunction initiated by alterations in cardiac energy substrates in the absence of coronary artery disease and hypertension. In addition to the demonstrated burden of cardiovascular events associated with diabetes, diabetic cardiomyopathy partly explains why diabetic patients are subject to a greater risk of heart failure and a worse outcome after myocardial ischemia. The raising prevalence and accumulating costs of cardiovascular disease in diabetic patients underscore the deficiencies of tertiary prevention and call for a shift in medical treatment. It is becoming increasingly clearer that the effective prevention and treatment of diabetic cardiomyopathy require measures to regulate the metabolic derangement occurring in the heart rather than merely restoring suitable systemic parameters. Recent research has provided deeper insight into the metabolic etiology of diabetic cardiomyopathy and numerous heart-specific targets that may substitute or reinforce current strategies. From both experimental and translational perspectives, in this review we first discuss the progress made with conventional therapies, and then focus on the need for prospective metabolic targets that may avert myocardial vulnerability and functional decline in next-generation diabetic care.

## Introduction

As of 2013, one in ten individuals living in Western countries was diagnosed with diabetes mellitus (DM), and three in ten were obese [1]. Epidemiological studies have long recognized diabetes to be an independent risk factor for heart failure (HF) and a worse prognosis after myocardial infarction (MI) [2]. Further, imaging studies have shown that diabetic patients are prone to ventricular hypertrophy, diastolic dysfunction, and decreased myocardial strain.

Diabetic cardiomyopathy (DCM) encompasses the group of diabetic patients who, while being otherwise free of cardiovascular events, display echocardiographic abnormalities ranging from mild diastolic impairment to overt heart failure, and prevalence of DCM may be over 50% among patients in clinical and preclinical stages [3,4]. Triggered by insulin inefficiency, hyperglycemia and hyperlipidemia, DCM brings out the damage of the myocardium due to cell hypertrophy, steatosis, apoptosis, oxidative stress, inflammation, fibrosis, and microtubular derangement [5,6]. The existence of cardiac sinusoids representing fetal vascular pattern support that the pathophysiological mechanism of DCM initiates in the coronary microcirculation due to alterations in cardiac metabolism [6]. Although this phenotype has been described in both type-I (T1DM) and type-II (T2DM) diabetic patients, hyperglycemia-induced myocardial fibrosis may be predominant in T1DM hearts, while cardiomyocyte hypertrophy and steatosis are more related to the insulin resistance and dyslipidemia of T2DM [7]. In addition, the earlier onset of ventricular remodelling and down-regulation of pro-survival factors in experimental female rats but not yet in male counterparts suggests gender differences in DCM progression [8].

Intensive glycemic goals (HbA1c <7%) have failed to prevent cardiac complications in long-term diabetic patients or have even increased cardiovascular mortality [9]. The failure of the therapy approaches employed to date calls for accelerating efforts towards the development of new therapeutic strategies capable of preserving heart function while contributing to the overall care of diabetes. Growing experimental research emphasizes the prospective utility of correcting the metabolic imbalance that occurs in the diabetic heart.

In this work we aim to provide an updated, metabolism-oriented overview of the main therapeutic findings on therapy approaches for DCM. A comprehensive search of the PubMed/MEDLINE and EMBASE databases was conducted for articles published up to and including 2014 using the terms “diabetic cardiomyopathy” and “diabetes heart” in combination with the different animal models, drugs and related pathogenic factors to retrieve available literature. All types of articles (e.g., original studies, randomized controlled trials, review articles) published in English and reporting on studies in animals and humans were included.

### Pathobiology of diabetic cardiomyopathy

Chronic hyperglycemia is primarily elicited by insulin deficiency and/or resistance and damages the myocardium by reducing the expression of glucose transporters, by increasing the expression of the pyruvate decarboxylase inhibitor pyruvate dehydrogenase kinase-4 (PDK4), and by raising the flux of glucose into hexosamine, pentose and polyol pathways. Such metabolic changes increase the burden of advanced glycation end-products (AGEs) and restrict energy production to fatty acid (FA) [5]. The harmful effects of AGEs are related to their ability to either promote collagen cross-linking and myocardial fibrosis or activate AGE receptor (RAGE)-mediated oxidative stress and pro-inflammatory nuclear factor-κB (NF-κB) signaling. Also, derangement of lipid metabolism is widely accepted as the most deleterious impact of diabetes on the heart. FA uptake may exceed the capacity for cardiac utilization and promote the deposition of triacylglycerols (TAGs) and ceramides, thus leading to cardiomyocyte steatosis and hypertrophy. Concurrently, increased FA oxidation leads to higher rates of oxygen consumption, reactive oxygen and nitrogen species (ROS/RNS) overproduction, endoplasmic reticulum (ER) stress and mitochondrial uncoupling [5]. Subsequent mitochondrial damage and increased ROS seem to be the main triggers for calcium mishandling, cardiomyocyte apoptosis, and the chronic low-grade inflammation occurring in DCM [10,11]. Moreover, the pro-oxidant status may be accentuated by defects in copper trafficking leading to myocardial copper deficiency [12]. All these pathogenic factors, in conjunction with many others (for further detail, see [5]) eventually converge to generate the progressive impairment of ventricular contractility, and thus provide valuable therapeutic substrates for the diabetic heart.

### Progress and limitations of conventional antihyperglycemic therapies

DCM may account for a substantial fraction of idiopathic HF diagnoses in T2DM patients, and also contribute to a worse prognosis of HF after myocardial ischemia in these patients. The United Kingdom Prospective Diabetes Study 34 (UKPDS 34) evaluated the outcomes of overweight T2DM patients following tight glycemic control with metformin, and is the only clinical study to show reduced cardiovascular mortality following intensive blood-glucose control [13]. However, there is long-standing controversy regarding the safety and efficacy of metformin in the setting of HF. A recent systematic review of observational studies involving 34,000 patients concluded that metformin is at least as safe as other glucose-lowering treatments even in the presence of reduced ejection fraction and chronic kidney disease [14]. Ongoing trials will soon provide specific evidence on the role of metformin in T2DM patients with HF (e.g. NCT01690091 - ClinicalTrials.gov). A variety of preclinical studies have pointed out the cardioprotective benefits of metformin on both T1DM and T2DM animal models of DCM, an effect likely associated with its ability to prevent cardiomyocyte oxidative stress and apoptosis by inhibiting the respiratory complex I and modulating specific ER stress signaling pathways (Table 1) [15-22]. Yet, the positive outcome on T1DM models seems more related to a dramatic reduction of plasma glucose and TAGs than to a myocardial-specific response (Table 2) [15]. Although metformin is not prescribed for most T1DM patients, it is widely recommended as the first-line oral therapy for T2DM. Unexpectedly, in T2DM animals cardiac fibrosis and contractile performance together with molecular markers of inflammation were not consistently improved or even aggravated. Such effects challenge our knowledge of the impact on metformin in the heart and question its utility against DCM. Of other conventional antihyperglycemic agents, only sulfonylureas have been proven to exert some benefit against experimental DCM (Table 1) [23]. However, sulfonylureas have been associated with an excess risk of MI and cardiovascular events [13], with the only possible exception of gliclazide [24]. Further, the use of sulfonylureas has been clearly restricted given their tendency to trigger hypoglycemias and weight gain (Table 2).

**Table 1:** **Progress on the effects of conventional metabolic therapies in experimental DCM**

	**Type 1 diabetes**			**Type 2 diabetes**				
	**STZ**	**OVE26**	**Akita**	***ob/ob***	***db/db***	**ZDF**	**GK**	**DIO** ^**†**^
Metformin	↑Glucose utilization ↑Cardiac function	↓Apoptosis ↑Cardiac function		↓Hypertrophy ~ Fibrosis ↑Cardiac function		~Hypertrophy ↑Fibrosis ↓Steatosis	↓/~Hypertrophy ↓Fibrosis ↓Apoptosis ~ Oxidative stress ↑Cardiac function	
Sulfonylureas	↑Cardiac function							
DPP-4 inhibitors			↑Hypertrophy ↑Cardiac function		~Hypertrophy ↓Fibrosis ↓Oxidative stress ~Cardiac function		↓Hypertrophy ↓Fibrosis ↓Apoptosis ↑Cardiac function	
GLP-1R agonists								↑Glucose utilization ↓Hypertrophy ↓Apoptosis ↓Inflammation ↑Cardiac function
Statins	↓Fibrosis ↓Oxidative stress ↓Inflammation ↑Cardiac function							↓Hypertrophy ↓Fibrosis ↓Inflammation ↑Cardiac function
PPARα agonists					↑Glucose utilization ↓Steatosis ~ Cardiac function	~Hypertrophy ↓Fibrosis ↓Steatosis		↑Glucose utilization ↓ER stress ↓Inflammation ↑Cardiac function
PPARγ agonists	↓Hypertrophy ↓Apoptosis ↓Steatosis ↓Oxidative stress ↑Cardiac function		↑Hypertrophy		↑Glucose utilization ~ Cardiac function	↑Glucose utilization		
**Reference**	[15,16,23,25-27]	[17]	[28,29]	[18]	[30-32]	[19,33]	[20,21]	[34-38]

Conventional treatments against DCM have been assessed in different animal models of type-I [STZ, streptozotocin-treated mice/rats; OVE26, calmodulin transgenic mice; Akita, insulin-2 deficient mice] and type-II [*ob/ob*, leptin deficient mice; *db/db*, leptin receptor deficient mice; ZDF, Zucker Diabetic Fatty, heterozygous leptin receptor deficient rats; GK, Goto-Kakizaki rats; DIO, diet-induced obesity] diabetes. ↑, ↓ and ~ stand for increased, decreased or not modified effect, respectively. ^†^For the sake of simplicity, evidence from low-dose STZ- plus diet-induced T2DM models are displayed in the DIO column. References: [34] and [37].

**Table 2:** **Limitations and drawbacks for the use of conventional metabolic therapies against DCM**

**Therapy**	**Limitation/Drawback**	**Reference**
Metformin	Dependence on plasma glucose reduction	[15,19]
	Inconsistent recovery of cardiac function	
	Potential induction of myocardial fibrosis	
Sulfonylureas	Higher risk of total cardiovascular events (*except gliclazide*)	[13,23]
	Hypoglycemia	
	Weight gain	
DPP-4 inhibitors	Higher risk of subclinical cardiac dysfunction and HF hospitalization	[28,30,39,40]
	Inconsistent recovery of cardiac function and remodelling	
	Higher risk of myocardial hypertrophy with effective dose	
GLP-1R agonists	Lack of data from large clinical trials on cardiovascular outcome	
Statins	Dependence on plasma lipid reduction and vascular remodelling	[41,42]
	Adverse impact on insulin production and sensitivity	
	Higher risk of total cardiovascular events (*torcetrapib*)	
PPARα agonists	Dependence on plasma lipid reduction	[31,38,43]
	Inconsistent recovery of cardiac function (*fenofibrate*)	
	DCM-like phenotype by experimental PPARα agonism	
PPARγ agonists	Dependence on plasma lipid reduction	[29,44,45]
	Higher risk of HF (*pioglitazone*)	
	Higher risk of total cardiovascular events (*rosiglitazone*)	
	Inconsistent recovery of cardiac function (*rosiglitazone*)	
	Potential induction of myocardial hypertrophy (*rosiglitazone*)	
	DCM-like phenotype by experimental PPARγ agonism	

### New directions in therapy against glucose-associated injury

Current trends in the care of the diabetic heart have downgraded the importance of HbA1c and other classical glycemic markers. However, abrogation of myocardial-specific insulin resistance and glucose toxicity could still be a valid strategy against DCM. Some of the most intuitive genetic interventions with therapeutic interest have targeted GLUT4, the most prevalent insulin-regulated glucose transporter in the heart, PDK4 and insulin-like growth factor-1 (IGF-1) (Table 3) [46-48]. Yet, these approaches face the potential threat of inducing intracellular glucose excess and associated toxicity. In this scenario, incretins may be advantageous. Although drugs modulating the incretin system are conventional agents in the management of diabetes, they may have more cardioprotective properties than those currently known (Table 1) [20,28,30,34-36,49]. The cardinal role of incretins (i.e. glucagon-like peptide 1, GLP-1) is the regulation of postprandial glycemia by stimulating insulin secretion in a glucose-dependent manner. Such endogenous modulation provides an exciting advantage over conventional antihyperglycemic drugs. Moreover, GLP-1 receptor (GLP-1R) has been detected in large amounts in the sarcolemma of isolated cardiomyoctes, thus suggesting that the cardiac effects of GLP-1 may be partially driven in a myocardial, insulin-independent, and specific fashion [20]. GLP-1 has been shown to enhance glucose uptake and limit FA consumption and lipotoxicity, likely by increasing GLUT-1 translocation and down-regulating the main cardiac FA transporter FAT/CD36 [30,50,51]. Further, we described anti-apoptotic/fibrotic effects of exogenous GLP-1 and its insulinotropic inactive metabolite GLP-1(9–36) in cardiomyocytes under diabetic conditions [20]. The first set of drugs to be developed, dipeptidyl peptidase-4 (DPP-4) inhibitors, increases GLP-1 levels in a physiological range and are weight neutral (Table 1). However, palliation of cardiac architectural and functional alterations was inconsistent among different models of T2DM exposed to sitagliptin [20,28,30]. In addition, the Saxagliptin Assessment of Vascular Outcomes Recorded in Patients with Diabetes Mellitus (SAVOR)-Thrombolysis in Myocardial Infarction (TIMI) 53 (SAVOR-TIMI 53) trial reported a 27% increase in hospitalization due to HF among diabetic patients who received saxagliptin as compared with placebo, and found no significant reductions on any macrovascular endpoint [39]. No clear explanation has been provided for these heterogeneous results. In this regard, DPP-4 inhibitors may increase the circulating half-life of DPP-4, which has been independently associated with a higher risk of cardiac dysfunction in T2DM patients [40]. This may be explained by the ability of DPP-4 to inactivate non-incretin molecules such as the brain natriuretic peptide, several chemokines (i.e., SDF1), and energy-related molecules (i.e., peptide-YY) [52]. Further, dosage might also be a relevant drawback for at least the use of sitagliptin, given that the improvement of glycemic control required higher doses which in turn induced cardiac hypertrophy in experimental models (Table 2) [28]. Despite the disappointing results, DPP-4 inhibition has also elicited positive myocardial outcomes and thus further research on its cardioprotective actions is warranted. For instance, sitagliptin has been shown to increase myocardial glucose uptake and ejection fraction in cohorts of non-diabetic patients with non-ischemic cardiomyopathy and stable coronary artery disease, respectively [53,54].

**Table 3:** **Genetic manipulation of key metabolic mediators of DCM with prospective therapeutic relevance**

**Mechanism**	**Model**	**Year**	**Genetic engineering**	**Myocardial outcome**	**Reference**
Glucose-associated injury	*db/db*	2002	Transgenic GLUT4	↑Glucose utilization, ↑Cardiac function	[46]
	DIO	2011	Transgenic PDK4	↓Steatosis	[47]
	STZ	2001	Transgenic IGF-1	↓Hypertrophy, ↓Apoptosis, ↓Oxidative stress, ↑Cardiac function	[48]
	STZ	2009	RAGE knockdown	↑Mitochondrial function, ↑Cardiac function	[55]
	STZ	2013	Transgenic GLO-1	↓Fibrosis, ↓Oxidative stress, ↓Inflammation	[56]
	STZ	2005	Transgenic O-GlcNAcase	↑Ca^2+^ mobilization, ↑Cardiac function	[57]
	MHC-PPARγ	2010	PPARα knockout	↓Hypertrophy, ↓ Apoptosis, ~Steatosis, ↓Oxidative stress, ↑Mitochondrial function, ↑Cardiac function	[58]
Lipid-associated injury	DIO	2007	Transgenic PPARβ/δ	↓Steatosis	[59]
	MHC-PPARα	2007	FAT/CD36 knockout	↓Steatosis, ↑Glucose utilization, ~Hypertrophy, ↑Ca^2+^ mobilization, ↑Cardiac function	[60]
	MHC-PPARα	2010	LPL knockout	↓Hypertrophy, ↓Steatosis, ↑Glucose utilization, ↑Mitochondrial function, ↑Cardiac function	[61]
	STZ	2014	Perilipin-5 knockout	↓Steatosis, ↓Oxidative stress, ↑Cardiac function	[62]
	Akita	2013	Transgenic ATGL	↓Steatosis, ↑Glucose utilization, ↑Mitochondrial function, ↑Cardiac function	[63]
	DIO	2014	Transgenic ATGL	↓Steatosis, ↑Diastolic/Systolic function	[64]
	STZ	2008	Transgenic HSL	↓Fibrosis, ↓Steatosis, ↓Oxidative stress	[65]
	STZ	2014	Arachidonate 12/15-lypoxygenase	↓Fibrosis, ↓Oxidative stress, ↓Inflammation	[66]
	DIO	2012	Transgenic SCD1	↑Steatosis, ↑Glucose utilization, ↓Oxidative stress, ↓Apoptosis	[67]
	MHC-PPARγ	2012	Transgenic DGAT-1	~Steatosis, ↑Glucose utilization, ↑Cardiac function	[68]
	MHC-ACS	2009	Transgenic DGAT-1	↑Steatosis, ↓Apoptosis, ↑Glucose utilization, ↓Oxidative stress, ↑Mitochondrial function, ↑Cardiac function	[69]
	DIO	2014	Transgenic Adiponectin R1	↓Hypertrophy, ↓Steatosis, ↓Oxidative stress	[70]
	DIO	2013	Transgenic APPL1	↓Steatosis, ↑Glucose utilization, ↑Cardiac function	[71]
	MHC-LPL^GPI^	2004	Transgenic human apoB	↓Steatosis, ↑Glucose utilization	[72]
Metabolism-associated inflammation	STZ	2010/13	TLR4 knockdown	↓Hypertrophy, ↓Fibrosis, ↓Apoptosis, ↓Oxidative stress, ↑Cardiac function	[73,74]
	NOD	2012	TLR4 knockout	↓Steatosis, ↑Cardiac function	[75]
	STZ	2014	HMGB1 knockdown	↓Hypertrophy, ↓Fibrosis, ↑Cardiac function	[76]
	DIO + STZ	2013	NLRP3 knockdown	↓Hypertrophy, ↓Fibrosis, ↑Cardiac function	[37]

↑, ↓ and ~ stand for increased, decreased or not modified effect, respectively.

GLP-1R agonists may withstand some of the controversy concerning DPP-4 inhibitors (Table 1). Exenatide was recently shown to ameliorate heart performance by increasing glucose uptake and, consistent with Randle’s hypothesis, down-regulating FAT/CD36 in a DCM-like model [49]. Similarly, liraglutide improved cardiac outcome in diet- plus streptozotozin (STZ)-induced T2DM animals independent of restoration of fasting blood glucose [34]. Nevertheless, the mechanisms underlying such functional recovery remain elusive and the attenuation of molecular markers of myocardial remodeling still requires further support from histological examination [35,36]. In the clinical setting, exenatide achieved greater benefits in glycemic control, bodyweight and heart pressure as compared to sitagliptin or pioglitazone when all three were used in combination with metformin [77]. It is expected that the Liraglutide Effect and Action in Diabetes: Evaluation of Cardiovascular Outcome Results (LEADER) trial will for the first time provide insight into the cardiovascular impact of GLP-1R-agonists in T2DM patients. To fully assess the potential utility of incretin-based drugs against DCM, along with their safe contribution to the overall care of diabetes, more evidence is needed on its clinical use and a deeper understanding of the direct impact on cardiomyocytes must be attained.

Decreasing the excess of harmful AGEs is also a main goal of glycemic control in DCM. AGEs are well-described mediators of hyperglycemia-associated cellular injury caused by increased reactive substrate availability. Although there is clinical evidence that AGE disruption may improve diastolic impairment [78], no data on such an effect in DCM patients are available. Consistently, RAGE ablation or cross-linking disruption in STZ-treated animals has been shown to effectively revert cardiac dysfunction [55,79]. Main strategies to palliate AGE-induced dysfunction are depicted in Table 3 [56,57] and may make the case for targeting AGEs for prevention of DCM. Overall, facilitating glucose transport across the sarcolemma in a glucose-dependent manner and avoiding its conversion into toxic alternate products may mark a major success against DCM.

### Progress and limitations of conventional antihyperlipidemic therapies

The unsuccessful prevention of cardiac complications in long-term T2DM patients by glycemia-oriented therapies has prompted a shift in the care of the diabetic heart. Various clinical studies have reported an outstanding decrease of cardiovascular risk in T2DM patients who undergo intensive lipidemic control [80-82]. It is known that hyperlipidemia may lead to FA accumulation and lipotoxicity in the diabetic myocardium. Therapeutic standards for diabetic patients include the prescription of lipid modifying agents, mainly HMG-CoA reductase inhibitors (i.e., statins) and peroxisome proliferator-activated receptor (PPAR) agonists (i.e., fibrates, glitazones), in both primary and secondary prevention. While statins are one of the most prescribed drugs worldwide due to their effective and safe impact on cholesterol levels, they do not seem to affect the natural history of DCM and their repercussion on the diabetic myocardium is currently being investigated. Most animal evidence is based on STZ-treated animals, and it has been shown that a variety of statins successfully reverted the DCM phenotype (Table 1) [25,26,37]. Yet it remains unclear whether the improvement of cardiac performance is due mostly to decreased vascular remodeling and heart workload rather than to a direct cardiac effect. Major clinical shortcomings in need of explanation include the recently reported adverse impact of statins on insulin sensitivity and β-cell function [41] and the increase of overall cardiovascular events induced by other cholesterol-modulating therapies such as the HDL raiser torcetrapib (Table 2) [42]. Nevertheless, current data do not support discontinuing statin therapy in diabetic patients [41].

PPARs are critical regulators of lipid transport, storage and utilization mainly by inducing the expression of FAT/CD36, FA oxidation enzymes, and PDK4. Importantly, PPARs are differentially located in organic tissues: PPARα is abundant in the heart while PPARγ is preferentially found in the adipose tissue. In diabetic animals (Table 1), pharmacological agonism of PPARα or PPARγ alleviated the DCM phenotype, likely by normalizing myocardial insulin-stimulated glucose uptake and reducing myocardial FA misuse and accumulation [19,27,31-33,38]. However, most studies in animal models do not provide consistent evidence that these drugs can rescue cardiac contractile function, or whether the cardiac benefits are rather a partial consequence of their lipid-lowering action (Table 2) [27,29,31,32,38]. From a clinical perspective, the relative safety of widely prescribed fibrates contrasts with the fact that PPARγ-based strategies have faced important drawbacks. Fibrates have proved to reduce the incidence of non-fatal MI and cardiovascular deaths in diabetic patients [43], although their contribution was milder than expected and further effort is needed to elucidate their mechanistic rationale. The Prospective Pioglitazone Clinical Trial in Macrovascular Events (PROactive) observed reduced total cardiovascular events, but detected an increase in HF episodes [83]. Rosiglitazone, however, was associated with a two-fold increase of incidence of cardiovascular events as compared with metformin, which eventually led to its withdrawal from the market [44]. Consistent with their role as transcriptional regulators of lipid metabolism, the transgenic enhancement of both PPARα and PPARγ induced a DCM-like phenotype [58,84]. Such effects have been explained by the reciprocal repression of glucose uptake and utilization in a setting of exacerbated lipid use, and may underlie the harmful response to the aforementioned PPARγ agonists. Taken as a whole, PPARα and PPARγ agonisms do not seem to cause a clinically meaningful benefit on the cardiac outcome of T2DM patients, and may even aggravate the condition.

### New directions against lipid-associated injury

The critical role of PPARs in the regulation of lipid metabolism should not be overlooked, and further research on different isoforms may suggest future tools against DCM (Table 3). PPARβ/δ is the most prevalent isoform in the myocardium and, unlike PPARα, its expression level is not increased in experimental T2DM [59]. Both genetic manipulation and selective agonism in mouse hearts and human cardiac cells provided evidence of the therapeutic potential of PPARβ/δ against cardiac steatosis, inflammation and dysfunction [59,85,86]. Such effects may depend on the ability of PPARβ/δ to up-regulate FA oxidation enzymes and GLUT4 while limiting FA uptake and TAG synthesis [59]. Experimental PPARβ/δ activation also ameliorated dyslipidemia and insulin resistance and prevented the development of obesity [87]. Thus, it is plausible that PPARβ/δ-based drugs provide an advantage over current PPARα agonists in the treatment of the diabetic heart.

Aside from PPARs, several molecular targets are found modulating lipid metabolism. The genetic manipulation of molecules involved in the initial steps of lipid absorption and accumulation has revealed cardioprotective properties and suggested potential strategies against DCM (Table 3) [60-62]. From this group, FAT/CD36 is the most studied molecule and currently has aroused the highest expectations due to its crucial role in lipotoxicity [88]. Although FAT/CD36 may be responsible for up to 60% of cardiac FA uptake, the mechanism of FA trafficking across the sarcolemma is only partially understood. Similar to GLUT4, FAT/CD36 molecules are found stored inside endosomal vesicles and can be translocated upon stimulation by insulin and by contraction. The redistribution of FAT/CD36 to the sarcolemma is one of the earliest alterations occurring in the heart during diet-induced diabetes, and has been observed in ZDF rats and in STZ mice in proportion to the severity of insulin deficiency [89,90]. Consistently, ablation of FAT/CD36 rescued a PPARα over-expression-induced DCM phenotype [60]. The feasibility of modulating FAT/CD36 was recently addressed by Glatz et al. in diabetic cardiomyocytes, indicating the benefit of interfering with FAT/CD36 trafficking [91]. Interestingly, FAT/CD36 was recently shown to be down-regulated by sitagliptin in *db/db* mice [30], thus suggesting that myocardial lipid uptake could also depend upon GLP-1R signaling. Therefore, modulation of FAT/CD36 may deserve further attention as a prospective etiological treatment for the diabetic heart.

FAT/CD36 interference may also yield interesting effects on metabolism-associated inflammation given its role as Toll-like receptor-4 (TLR4) cofactor [92]. TLRs are pattern recognition receptors implicated in tailoring the innate immune cascade through conserved inflammatory pathways. Major cardiac isoforms, TLR4 and TLR2 represent an emerging standpoint for inflammatory and metabolic coupling in the diabetic heart, where they function as upstream inducers of NF-κB and regulate insulin resistance. Notably, ablation of TLR4 and associated ligands successfully reverted architectural aberrations and restored cardiac dysfunction in STZ-treated and non-obese T1DM (NOD) mice (Table 3) [73-76]. Moreover, pharmacological intervention of TLR4 has already caused a benefit in several forms of non-diabetic myocardial dysfunction [93]. It is also known that FAT/CD36 cooperates with TLR4 to prime the inflammasome, a group of multimeric protein complexes of which Nod-like receptor-3 (NLRP3) is the most common form, leading to caspase-1-mediated IL-1β processing and release [94,95]. Besides contributing to multiple pro-inflammatory and pro-fibrotic pathways, NLRP3 has also been shown to promote insulin resistance. Consistently, NLRP3 knockdown reduced cardiac hypertrophy and fibrosis and restored cardiac function in STZ-induced T2DM mice (Table 3) [37]. This emerging inflammatory perspective of FAT/CD36 actions reinforces the expectations for its targeting against DCM.

Genetic engineering has uncovered numerous other key molecules of lipid metabolism with potential use against DCM. This group includes rate-limiting lipases of TAG hydrolysis [63-65], arachidonate lypoxygenases [66], and mitochondrial enzymes involved in FA synthesis [67-69]. A practical summary of their impact in several animal models of DCM is shown in Table 3. There is also growing interest for the pharmacological utility of specific peptides regulating energy homeostasis such as adiponectin and ghrelin. In this regard, genetic enhancement of upstream components of adiponectin signalling ameliorated DCM phenotype, glucose uptake and cardiac function in diet-induced T2DM [70,71]. Similar results were recently described in *db/db* mice administered desacyl ghrelin, the non-octanoylated form of this “hunger hormone” [96]. Remarkably, the intervention of some of these factors achieved functional recovery despite inconsistent lowering of cardiac TAG content. In sum, since the increase of FA oxidation is tightly coupled with higher rates of oxygen consumption and ROS/RNS generation, it would seem reasonable to assume that therapies against lipid uptake will provide advantage over those enhancing intracellular metabolism. We would also argue that prospective therapies should not focus on reducing the content of cardiac TAGs but rather prioritize the burden of derived molecules with greater toxicity such as diacylglycerides and ceramides.

## Conclusions

**Figure 1:**
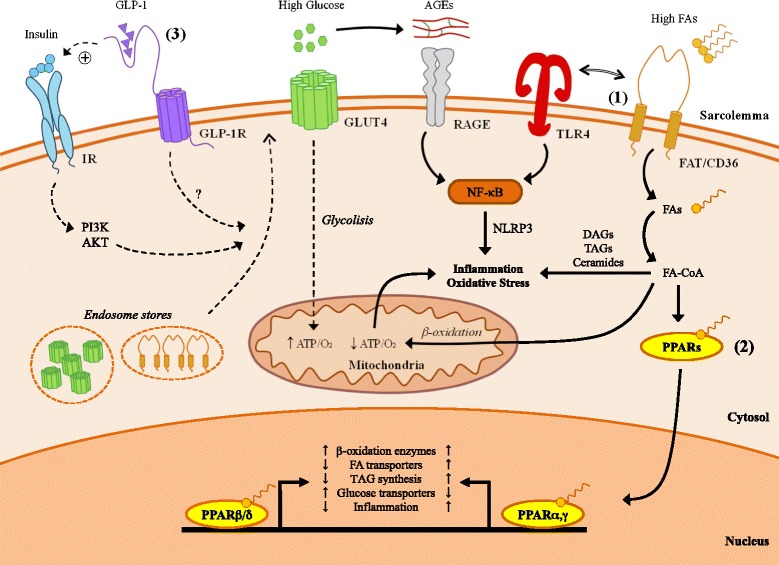
**Metabolic disturbance in the diabetic heart and prospective therapeutic targets.** Thickened lines denote activated pathways, and dotted lines denote reduced pathway. **(1)** The therapeutic reduction of FAT/CD36 activity may attenuate myocardial steatosis, inflammation and oxidative stress, and further improve the energetic yield by shifting metabolism to glucose utilization. **(2)** Induction of specific PPAR isoforms such as PPARβ/δ may provide cardioprotection by down-regulating FA transporters and TAG synthesis and up-regulating GLUT4, β-oxidation enzymes, and anti-inflammatory transcripts. **(3)** The elevation of incretin signaling by GLP-1 agonists (or DPP-4 inhibitors) may also control insulin resistance and hyperlipidemia. GLP-1R-dependent actions may include the regulation of glucose and FA receptors trafficking to the sarcolemma, and the amelioration of apoptosis and fibrosis. IR, insulin receptor; FA-CoA, fatty acid-coenzyme A.

As therapeutic resources currently allow diabetic patients to manage glucose homeostasis, reach near-normal life expectancy and delay microvascular complications, it is unacceptable that heart-risk reductions are not yet properly achieved. By targeting DCM we may prevent cardiac functional decline and improve the response to potentially lethal coronary events. Although the fine-tune mechanisms leading to DCM remain partially opaque, restoring cardiac energy metabolism seem to be a cornerstone. In light of the experimental data provided here, we may outline a potential therapeutic strategy focused on correcting the etiological imbalance between lipids and glucose as fuel substrates (Figure 1). Excessive FA uptake, accumulation, and utilization may be attenuated by down-modulating cardiac FAT/CD36 and/or stimulating its transcriptional regulator PPARβ/δ. Subsequent up-regulation of GLUT4 and glycolitic enzymes would elevate the ATP/O_2_^-^consumption ratio and reduce hyperglycemia and AGE generation. Insulin resistance and hyperlipidemia may be also controlled by statins, and perhaps more prospectively by incretin-based drugs, which would balance the content of sarcolemmal glucose and FA transporters and exert anti-apoptotic/fibrotic benefits in the cardiomyocyte. In addition, FAT/CD36 interference may provide further protection against myocardial inflammation and stiffness through the attenuation of TLR4-NLRP3 signaling. The design of these cardioprotective plans, however, may lead to difficulties concerning the possibility of provoking other end-organ complications associated with glucotoxicity. To overcome these hurdles, the assessment of differential traits, stages and pathogenic factors in each diabetic patient becomes a clinical priority.

## Abbreviations

DM: Diabetes mellitus
HF: Heart failure
DCM: Diabetic cardiomyopathy
T1DM/T2DM: Type 1 or Type 2 Diabetes Mellitus
PDK4: Pyruvate dehydrogenase kinase-4
AGEs: Advanced glycation end-products
FA: Fatty acid
RAGE: AGE receptor
NF-κB: Nuclear factor-κB
TAGs: Triacylglycerols
ROS/RNS: Reactive oxygen and nitrogen species
IGF-1: Insulin-like growth factor-1
GLP-1: Glucagon-like peptide-1
DPP-4: Dipeptidyl peptidase-4
PPAR: Peroxisome proliferator-activated receptor
TLR4: Toll-like receptor-4
NLRP3: Nod-like receptor-3
HMGB1: High-mobility group box-1

## References

[1] Diabetes Atlas | International Diabetes Federation. [http://www.idf.org/diabetesatlas]

[2] Bell DSH (2003). Diabetic Cardiomyopathy. Diabetes Care.

[3] Poirier P, Bogaty P, Garneau C, Marois L, Dumesnil JG (2001). Diastolic dysfunction in normotensive men with well-controlled type 2 diabetes: importance of maneuvers in echocardiographic screening for preclinical diabetic cardiomyopathy. Diabetes Care.

[4] Kiencke S, Handschin R, von Dahlen R, Muser J, Brunner-Larocca HP, Schumann J (2010). Pre-clinical diabetic cardiomyopathy: prevalence, screening, and outcome. Eur J Heart Fail.

[5] Bugger H, Abel ED (2014). Molecular mechanisms of diabetic cardiomyopathy. Diabetologia.

[6] Manjarrez-Gutiérrez G, Hernández-Chávez V, Neri-Gómez T, Boyzo-Montes de Oca A, Mondragón-Herrera JA, Hernández-Rodríguez J (2014). Anatomopathological findings during development of diabetic cardiomyopathy in rats. Cir Cir.

[7] Phillips RA, Krakoff LR, Dunaif A, Finegood DT, Gorlin R, Shimabukuro S (1998). Relation among left ventricular mass, insulin resistance, and blood pressure in nonobese subjects. J Clin Endocrinol Metab.

[8] Moore A, Shindikar A, Fomison-Nurse I, Riu F, Munasinghe PE, Ram TP (2014). Rapid onset of cardiomyopathy in STZ-induced female diabetic mice involves the downregulation of pro-survival Pim-1. Cardiovasc Diabetol.

[9] Mazzone T (2010). Intensive Glucose Lowering and Cardiovascular Disease Prevention in Diabetes Reconciling the Recent Clinical Trial Data. Circulation.

[10] Hamblin M, Friedman DB, Hill S, Caprioli RM, Smith HM, Hill MF (2007). Alterations in the diabetic myocardial proteome coupled with increased myocardial oxidative stress underlies diabetic cardiomyopathy. J Mol Cell Cardiol.

[11] Palomer X, Salvadó L, Barroso E, Vázquez-Carrera M (2013). An overview of the crosstalk between inflammatory processes and metabolic dysregulation during diabetic cardiomyopathy. Int J Cardiol.

[12] Zhang S, Liu H, Amarsingh GV, Cheung CCH, Hogl S, Narayanan U (2014). Diabetic cardiomyopathy is associated with defective myocellular copper regulation and both defects are rectified by divalent copper chelation. Cardiovasc Diabetol.

[13] Effect of intensive blood-glucose control with metformin on complications in overweight patients with type 2 diabetes (UKPDS 34). The Lancet. 1998;352:854–865. 9742977

[14] Eurich DT, Weir DL, Majumdar SR, Tsuyuki RT, Johnson JA, Tjosvold L et al. Comparative Safety and Effectiveness of Metformin in Patients with Diabetes and Heart Failure: Systematic Review of Observational Studies Involving 34000 Patients. Circ Heart Fail.2013;CIRCHEARTFAILURE.112.000162. 10.1161/CIRCHEARTFAILURE.112.000162 23508758

[15] Verma S, McNeill JH (1994). Metformin improves cardiac function in isolated streptozotocin-diabetic rat hearts. Am J Physiol.

[16] Da Silva D, Ausina P, Alencar EM, Coelho WS, Zancan P, Sola-Penna M (2012). Metformin reverses hexokinase and phosphofructokinase downregulation and intracellular distribution in the heart of diabetic mice. IUBMB Life.

[17] Xie Z, Lau K, Eby B, Lozano P, He C, Pennington B (2011). Improvement of cardiac functions by chronic metformin treatment is associated with enhanced cardiac autophagy in diabetic OVE26 mice. Diabetes.

[18] Kim J, Wietecha TA, Dai D-F, Sullivan B, Sta Teresa A, Hudkins KL (2012). Metformin Prevents the Development of Cardiomyopathy and Cardiac Dysfunction in Diabetogenic Diet-fed BTBRob/+ Mice (Abstract). Circulation.

[19] Forcheron F, Basset A, Abdallah P, Carmine PD, Gadot N, Beylot M (2009). Diabetic cardiomyopathy: effects of fenofibrate and metformin in an experimental model – the Zucker diabetic rat. Cardiovasc Diabetol.

[20] Picatoste B, Ramírez E, Caro-Vadillo A, Iborra C, Egido J, Tuñón J (2013). Sitagliptin reduces cardiac apoptosis, hypertrophy and fibrosis primarily by insulin-dependent mechanisms in experimental type-II diabetes. Potential roles of GLP-1 isoforms. PLoS One.

[21] Rösen P, Wiernsperger NF (2006). Metformin delays the manifestation of diabetes and vascular dysfunction in Goto-Kakizaki rats by reduction of mitochondrial oxidative stress. Diabetes Metab Res Rev.

[22] Quentin T, Steinmetz M, Poppe A, Thoms S (2012). Metformin differentially activates ER stress signaling pathways without inducing apoptosis. Dis Model Mech.

[23] Mozaffari MS, Allo S, Schaffer SW (1989). The effect of sulfonylurea therapy on defective calcium movement associated with diabetic cardiomyopathy. Can J Physiol Pharmacol.

[24] Patel A, MacMahon S, Chalmers J, Neal B, Billot L, Woodward M, ADVANCE Collaborative Group (2008). Intensive blood glucose control and vascular outcomes in patients with type 2 diabetes. N Engl J Med.

[25] Shida T, Nozawa T, Sobajima M, Ihori H, Matsuki A, Inoue H (2014). Fluvastatin-induced reduction of oxidative stress ameliorates diabetic cardiomyopathy in association with improving coronary microvasculature. Heart Vessels.

[26] Quidgley J, Cruz N, Crespo MJ (2014). Atorvastatin improves systolic function, but does not prevent the development of dilated cardiomyopathy in streptozotocin-induced diabetic rats. Ther Adv Cardiovasc Dis.

[27] Baraka A, AbdelGawad H (2010). Targeting apoptosis in the heart of streptozotocin-induced diabetic rats. J Cardiovasc Pharmacol Ther.

[28] Hemmeryckx B, Swinnen M, Gallacher DJ, Rong Lu H, Roger Lijnen H (2014). Effect of sitagliptin treatment on metabolism and cardiac function in genetic diabetic mice. Eur J Pharmacol.

[29] Hemmeryckx B, Hoylaerts MF, Gallacher DJ, Rong Lu H, Himmelreich U, D’hooge J (2013). Does rosiglitazone affect adiposity and cardiac function in genetic diabetic mice?. Eur J Pharmacol.

[30] Lenski M, Kazakov A, Marx N, Böhm M, Laufs U (2011). Effects of DPP-4 inhibition on cardiac metabolism and function in mice. J Mol Cell Cardiol.

[31] Aasum E, Belke DD, Severson DL, Riemersma RA, Cooper M, Andreassen M (2002). Cardiac function and metabolism in Type 2 diabetic mice after treatment with BM 17.0744, a novel PPAR-alpha activator. Am J Physiol Heart Circ Physiol.

[32] Carley AN, Semeniuk LM, Shimoni Y, Aasum E, Larsen TS, Berger JP (2004). Treatment of type 2 diabetic db/db mice with a novel PPARgamma agonist improves cardiac metabolism but not contractile function. Am J Physiol Endocrinol Metab.

[33] Sidell RJ, Cole MA, Draper NJ, Desrois M, Buckingham RE, Clarke K (2002). Thiazolidinedione treatment normalizes insulin resistance and ischemic injury in the zucker Fatty rat heart. Diabetes.

[34] Liu J, Liu Y, Chen L, Wang Y, Li J (2013). Glucagon-Like Peptide-1 Analog Liraglutide Protects against Diabetic Cardiomyopathy by the Inhibition of the Endoplasmic Reticulum Stress Pathway. J Diabetes Res.

[35] Noyan-Ashraf MH, Shikatani EA, Schuiki I, Mukovozov I, Wu J, Li R-K (2013). A glucagon-like peptide-1 analog reverses the molecular pathology and cardiac dysfunction of a mouse model of obesity. Circulation.

[36] Mells JE, Fu PP, Sharma S, Olson D, Cheng L, Handy JA (2012). Glp-1 analog, liraglutide, ameliorates hepatic steatosis and cardiac hypertrophy in C57BL/6J mice fed a Western diet. Am J Physiol - Gastrointest Liver Physiol.

[37] Luo B, Li B, Wang W, Liu X, Liu X, Xia Y, Zhang C, Zhang Y, Zhang M, An F (2014). Rosuvastatin Alleviates Diabetic Cardiomyopathy by Inhibiting NLRP3 Inflammasome and MAPK Pathways in a Type 2 Diabetes Rat Model. Cardiovasc Drugs Ther Spons Int Soc Cardiovasc Pharmacother.

[38] Aasum E, Khalid AM, Gudbrandsen OA, How O-J, Berge RK, Larsen TS (2008). Fenofibrate modulates cardiac and hepatic metabolism and increases ischemic tolerance in diet-induced obese mice. J Mol Cell Cardiol.

[39] Scirica BM, Bhatt DL, Braunwald E, Steg PG, Davidson J, Hirshberg B (2013). Saxagliptin and cardiovascular outcomes in patients with type 2 diabetes mellitus. N Engl J Med.

[40] Ravassa S, Barba J, Coma-Canella I, Huerta A, López B, González A (2013). The activity of circulating dipeptidyl peptidase-4 is associated with subclinical left ventricular dysfunction in patients with type 2 diabetes mellitus. Cardiovasc Diabetol.

[41] Barylski M, Nikolic D, Banach M, Toth PP, Montalto G, Rizzo M. STATINS AND NEW-ONSET DIABETES. Curr Pharm Des. 2013. 10.2174/13816128113196660678 24040871

[42] Barter PJ, Caulfield M, Eriksson M, Grundy SM, Kastelein JJP, Komajda M (2007). Effects of Torcetrapib in Patients at High Risk for Coronary Events. N Engl J Med.

[43] Keech A, Simes RJ, Barter P, Best J, Scott R, Taskinen MR (2005). Effects of long-term fenofibrate therapy on cardiovascular events in 9795 people with type 2 diabetes mellitus (the FIELD study): randomised controlled trial. Lancet.

[44] Komajda M, McMurray JJV, Beck-Nielsen H, Gomis R, Hanefeld M, Pocock SJ (2010). Heart failure events with rosiglitazone in type 2 diabetes: data from the RECORD clinical trial. Eur Heart J.

[45] Giles TD, Miller AB, Elkayam U, Bhattacharya M, Perez A (2008). Pioglitazone and heart failure: results from a controlled study in patients with type 2 diabetes mellitus and systolic dysfunction. J Card Fail.

[46] Semeniuk LM, Kryski AJ, Severson DL (2002). Echocardiographic assessment of cardiac function in diabeticdb/db and transgenic db/db-hGLUT4 mice. Am J Physiol - Heart Circ Physiol.

[47] Chambers KT, Leone TC, Sambandam N, Kovacs A, Wagg CS, Lopaschuk GD (2011). Chronic Inhibition of Pyruvate Dehydrogenase in Heart Triggers an Adaptive Metabolic Response. J Biol Chem.

[48] Kajstura J, Fiordaliso F, Andreoli AM, Li B, Chimenti S, Medow MS (2001). IGF-1 Overexpression Inhibits the Development of Diabetic Cardiomyopathy and Angiotensin II–Mediated Oxidative Stress. Diabetes.

[49] Liu L, Trent CM, Fang X, Son N-H, Jiang H, Blaner WS et al. Cardiomyocyte specific loss of diacylglycerol acyl transferase 1 (DGAT1) reproduces the abnormalities in lipids found in severe heart failure. J Biol Chem. 2014. 10.1074/jbc.M114.601864 PMC4207999 25157099

[50] Zhao T, Parikh P, Bhashyam S, Bolukoglu H, Poornima I, Shen Y-T (2006). Direct effects of glucagon-like peptide-1 on myocardial contractility and glucose uptake in normal and postischemic isolated rat hearts. J Pharmacol Exp Ther.

[51] Nagashima M, Watanabe T, Terasaki M, Tomoyasu M, Nohtomi K, Kim-Kaneyama J (2011). Native incretins prevent the development of atherosclerotic lesions in apolipoprotein E knockout mice. Diabetologia.

[52] Zhong J, Rao X, Rajagopalan S (2013). An emerging role of dipeptidyl peptidase 4 (DPP4) beyond glucose control: potential implications in cardiovascular disease. Atherosclerosis.

[53] Witteles RM, Keu KV, Quon A, Tavana H, Fowler MB (2012). Dipeptidyl peptidase 4 inhibition increases myocardial glucose uptake in nonischemic cardiomyopathy. J Card Fail.

[54] Read PA, Khan FZ, Heck PM, Hoole SP, Dutka DP (2010). DPP-4 inhibition by sitagliptin improves the myocardial response to dobutamine stress and mitigates stunning in a pilot study of patients with coronary artery disease. Circ Cardiovasc Imaging.

[55] Ma H, Li S-Y, Xu P, Babcock SA, Dolence EK, Brownlee M (2009). Advanced glycation endproduct (AGE) accumulation and AGE receptor (RAGE) up-regulation contribute to the onset of diabetic cardiomyopathy. J Cell Mol Med.

[56] Brouwers O, de Vos-Houben JMJ, Niessen PMG, Miyata T, van Nieuwenhoven F, Janssen BJA (2013). Mild oxidative damage in the diabetic rat heart is attenuated by glyoxalase-1 overexpression. Int J Mol Sci.

[57] Hu Y, Belke D, Suarez J, Swanson E, Clark R, Hoshijima M (2005). Adenovirus-mediated overexpression of O-GlcNAcase improves contractile function in the diabetic heart. Circ Res.

[58] Son N-H, Yu S, Tuinei J, Arai K, Hamai H, Homma S (2010). PPARγ-induced cardiolipotoxicity in mice is ameliorated by PPARα deficiency despite increases in fatty acid oxidation. J Clin Invest.

[59] Burkart EM, Sambandam N, Han X, Gross RW, Courtois M, Gierasch CM (2007). Nuclear receptors PPARbeta/delta and PPARalpha direct distinct metabolic regulatory programs in the mouse heart. J Clin Invest.

[60] Yang J, Sambandam N, Han X, Gross RW, Courtois M, Kovacs A (2007). CD36 deficiency rescues lipotoxic cardiomyopathy. Circ Res.

[61] Duncan JG, Bharadwaj KG, Fong JL, Mitra R, Sambandam N, Courtois MR (2010). Rescue of cardiomyopathy in peroxisome proliferator-activated receptor-alpha transgenic mice by deletion of lipoprotein lipase identifies sources of cardiac lipids and peroxisome proliferator-activated receptor-alpha activators. Circulation.

[62] Kuramoto K, Sakai F, Yoshinori N, Nakamura TY, Wakabayashi S, Kojidani T, Haraguchi T, Hirose F, Osumi T (2014). Deficiency of a lipid droplet protein, Perilipin 5, suppresses myocardial lipid accumulation, thereby preventing type 1 diabetes-induced heart malfunction. Mol Cell Biol.

[63] Pulinilkunnil T, Kienesberger PC, Nagendran J, Waller TJ, Young ME, Kershaw EE (2013). Myocardial Adipose Triglyceride Lipase Overexpression Protects Diabetic Mice From the Development of Lipotoxic Cardiomyopathy. Diabetes.

[64] Pulinilkunnil T, Kienesberger PC, Nagendran J, Sharma N, Young ME, Dyck JRB (2014). Cardiac-specific adipose triglyceride lipase overexpression protects from cardiac steatosis and dilated cardiomyopathy following diet-induced obesity. Int J Obes.

[65] Ueno M, Suzuki J, Zenimaru Y, Takahashi S, Koizumi T, Noriki S (2008). Cardiac overexpression of hormone-sensitive lipase inhibits myocardial steatosis and fibrosis in streptozotocin diabetic mice. Am J Physiol Endocrinol Metab.

[66] Suzuki H, Kayama Y, Sakamoto M, Iuchi H, Shimizu I, Yoshino T et al. Arachidonate 12/15-Lipoxygenase-Induced Inflammation and Oxidative Stress Are Involved in the Development of Diabetic Cardiomyopathy. Diabetes. 2014.Sep 3. 10.2337/db13-1896 25187369

[67] Matsui H, Yokoyama T, Sekiguchi K, Iijima D, Sunaga H, Maniwa M (2012). Stearoyl-CoA desaturase-1 (SCD1) augments saturated fatty acid-induced lipid accumulation and inhibits apoptosis in cardiac myocytes. PLoS One.

[68] Liu L, Yu S, Khan RS, Homma S, Schulze PC, Blaner WS (2012). Diacylglycerol acyl transferase 1 overexpression detoxifies cardiac lipids in PPARγ transgenic mice. J Lipid Res.

[69] Liu L, Shi X, Bharadwaj KG, Ikeda S, Yamashita H, Yagyu H (2009). DGAT1 Expression Increases Heart Triglyceride Content but Ameliorates Lipotoxicity. J Biol Chem.

[70] Chou I-P, Chiu Y-P, Ding S-T, Liu B-H, Lin YY, Chen C-Y (2014). Adiponectin receptor 1 overexpression reduces lipid accumulation and hypertrophy in the heart of diet-induced obese mice - possible involvement of oxidative stress and autophagy. Endocr Res.

[71] Park M, Wu D, Park T, Choi C, Li R-K, Cheng KKY (2013). APPL1 transgenic mice are protected from high-fat diet-induced cardiac dysfunction. Am J Physiol Endocrinol Metab.

[72] Yokoyama M, Yagyu H, Hu Y, Seo T, Hirata K, Homma S (2004). Apolipoprotein B Production Reduces Lipotoxic Cardiomyopathy studies in heart-specific lipoprotein lipase transgenic mouse. J Biol Chem.

[73] Haitao Z, Zhongwei L, Kunlun C, Xin D, Chuan Q, Dengfeng G (2013). ASSA13-06-6 Prevention of Cardiac Remodelling by Gene Silencing of Toll-Like Receptor-4 in Mice with Diabetic Cardiomyopathy. Heart.

[74] Zhang Y, Peng T, Zhu H, Zheng X, Zhang X, Jiang N (2010). Prevention of hyperglycemia-induced myocardial apoptosis by gene silencing of Toll-like receptor-4. J Transl Med.

[75] Dong B, Qi D, Yang L, Huang Y, Xiao X, Tai N (2012). TLR4 regulates cardiac lipid accumulation and diabetic heart disease in the nonobese diabetic mouse model of type 1 diabetes. Am J Physiol Heart Circ Physiol.

[76] Wang W-K, Wang B, Lu Q-H, Zhang W, Qin W-D, Liu X-J, Liu X-Q, An F-S, Zhang Y, Zhang M-X (2014). Inhibition of high-mobility group box 1 improves myocardial fibrosis and dysfunction in diabetic cardiomyopathy. Int J Cardiol.

[77] Bergenstal RM, Wysham C, Macconell L, Malloy J, Walsh B, Yan P (2010). Efficacy and safety of exenatide once weekly versus sitagliptin or pioglitazone as an adjunct to metformin for treatment of type 2 diabetes (DURATION-2): a randomised trial. Lancet.

[78] Little WC, Zile MR, Kitzman DW, Hundley WG, O’Brien TX, de Groof RC (2005). The Effect of Alagebrium Chloride (ALT-711), a Novel Glucose Cross-Link Breaker, in the Treatment of Elderly Patients With Diastolic Heart Failure. J Card Fail.

[79] Kranstuber AL, Del Rio C, Biesiadecki BJ, Hamlin RL, Ottobre J, Gyorke S (2012). Advanced glycation end product cross-link breaker attenuates diabetes-induced cardiac dysfunction by improving sarcoplasmic reticulum calcium handling. Front Physiol.

[80] Heart Protection Study Collaborative Group (2002). MRC/BHF Heart Protection Study of cholesterol lowering with simvastatin in 20,536 high-risk individuals: a randomised placebo-controlled trial. Lancet.

[81] Colhoun HM, Betteridge DJ, Durrington PN, Hitman GA, Neil HAW, Livingstone SJ (2004). Primary prevention of cardiovascular disease with atorvastatin in type 2 diabetes in the Collaborative Atorvastatin Diabetes Study (CARDS): multicentre randomised placebo-controlled trial. Lancet.

[82] Gæde P, Lund-Andersen H, Parving H-H, Pedersen O (2008). Effect of a multifactorial intervention on mortality in type 2 diabetes. N Engl J Med.

[83] Dormandy JA, Charbonnel B, Eckland DJA, Erdmann E, Massi-Benedetti M, Moules IK (2005). Secondary prevention of macrovascular events in patients with type 2 diabetes in the PROactive Study (PROspective pioglitAzone Clinical Trial In macroVascular Events): a randomised controlled trial. Lancet.

[84] Finck BN, Lehman JJ, Leone TC, Welch MJ, Bennett MJ, Kovacs A (2002). The cardiac phenotype induced by PPARalpha overexpression mimics that caused by diabetes mellitus. J Clin Invest.

[85] Palomer X, Capdevila-Busquets E, Botteri G, Salvadó L, Barroso E, Davidson MM (2014). PPARβ/δ attenuates palmitate-induced endoplasmic reticulum stress and induces autophagic markers in human cardiac cells. Int J Cardiol.

[86] Alvarez-Guardia D, Palomer X, Coll T, Serrano L, Rodríguez-Calvo R, Davidson MM (1811). PPARβ/δ activation blocks lipid-induced inflammatory pathways in mouse heart and human cardiac cells. Biochim Biophys Acta.

[87] Bedu E, Wahli W, Desvergne B (2005). Peroxisome proliferator-activated receptor beta/delta as a therapeutic target for metabolic diseases. Expert Opin Ther Targets.

[88] van de Weijer T, Schrauwen-Hinderling VB, Schrauwen P (2011). Lipotoxicity in type 2 diabetic cardiomyopathy. Cardiovasc Res.

[89] Luiken JJFP, Arumugam Y, Bell RC, Calles-Escandon J, Tandon NN, Glatz JFC (2002). Changes in fatty acid transport and transporters are related to the severity of insulin deficiency. Am J Physiol Endocrinol Metab.

[90] Coort SLM, Hasselbaink DM, Koonen DPY, Willems J, Coumans WA, Chabowski A (2004). Enhanced sarcolemmal FAT/CD36 content and triacylglycerol storage in cardiac myocytes from obese zucker rats. Diabetes.

[91] Glatz JFC, Angin Y, Steinbusch LKM, Schwenk RW, Luiken JJFP (2013). CD36 as a target to prevent cardiac lipotoxicity and insulin resistance. Prostaglandins Leukot Essent Fatty Acids.

[92] Stewart CR, Stuart LM, Wilkinson K, van Gils JM, Deng J, Halle A (2010). CD36 ligands promote sterile inflammation through assembly of a Toll-like receptor 4 and 6 heterodimer. Nat Immunol.

[93] Ehrentraut H, Weber C, Ehrentraut S, Schwederski M, Boehm O, Knuefermann P (2011). The toll-like receptor 4-antagonist eritoran reduces murine cardiac hypertrophy. Eur J Heart Fail.

[94] Sheedy FJ, Grebe A, Rayner KJ, Kalantari P, Ramkhelawon B, Carpenter SB (2013). CD36 coordinates NLRP3 inflammasome activation by facilitating the intracellular nucleation from soluble to particulate ligands in sterile inflammation. Nat Immunol.

[95] Fuentes-Antras J, Loan AM, Tuñón J, Egido J, Lorenzo O (2014). Activation of Toll-Like Receptors and Inflammasome Complexes in the Diabetic Cardiomyopathy-Associated Inflammation. Int J Endocrinol.

[96] Pei XM, Yung BY, Yip SP, Chan LW, Wong CS, Ying M, et al. Protective effects of desacyl ghrelin on diabetic cardiomyopathy. Acta Diabetol. 2014. Sept 6. 10.1007/s00592-014-0637-4 25192951

